# Probiotics: mechanism of action, health benefits and their application in food industries

**DOI:** 10.3389/fmicb.2023.1216674

**Published:** 2023-08-17

**Authors:** Anam Latif, Aamir Shehzad, Sobia Niazi, Asna Zahid, Waqas Ashraf, Muhammad Waheed Iqbal, Abdur Rehman, Tahreem Riaz, Rana Muhammad Aadil, Imran Mahmood Khan, Fatih Özogul, João Miguel Rocha, Tuba Esatbeyoglu, Sameh A. Korma

**Affiliations:** ^1^Department of Human Nutrition and Dietetics, School of Food and Agricultural Sciences, University of Management and Technology, Lahore, Pakistan; ^2^UniLaSalle, Univ. Artois, ULR7519 - Transformations & Agro-resources, Normandie Université, Mont-Saint-Aignan, France; ^3^State Key Laboratory of Food Science and Technology, Jiangnan University, Wuxi, China; ^4^National Institute of Food Science and Technology, University of Agriculture, Faisalabad, Pakistan; ^5^School of Food Science and Technology, Jiangnan University, Wuxi, Jiangsu, China; ^6^School of Food and Biological Engineering, Jiangsu University, Zhenjiang, Jiangsu, China; ^7^College of Food and Biological Engineering, Jimei University, Xiamen, China; ^8^Department of Seafood Processing Technology, Faculty of Fisheries, Cukurova University, Adana, Türkiye; ^9^Biotechnology Research and Application Center, Cukurova University, Adana, Türkiye; ^10^CBQF - Centro de Biotecnologia e Química Fina – Laboratório Associado, Escola Superior de Biotecnologia, Universidade Católica Portuguesa, Porto, Portugal; ^11^LEPABE—Laboratory for Process Engineering, Environment, Biotechnology and Energy, Faculty of Engineering, University of Porto, Porto, Portugal; ^12^ALiCE—Associate Laboratory in Chemical Engineering, Faculty of Engineering, University of Porto, Porto, Portugal; ^13^Department of Food Development and Food Quality, Institute of Food Science and Human Nutrition, Gottfried Wilhelm Leibniz University Hannover, Hannover, Germany; ^14^Department of Food Science, Faculty of Agriculture, Zagazig University, Zagazig, Egypt; ^15^School of Food Science and Engineering, South China University of Technology, Guangzhou, China

**Keywords:** probiotics, lactic acid bacteria, immunomodulation, anti-allergic and gastrointestinal diseases, functional foods

## Abstract

Probiotics, like lactic acid bacteria, are non-pathogenic microbes that exert health benefits to the host when administered in adequate quantity. Currently, research is being conducted on the molecular events and applications of probiotics. The suggested mechanisms by which probiotics exert their action include; competitive exclusion of pathogens for adhesion sites, improvement of the intestinal mucosal barrier, gut immunomodulation, and neurotransmitter synthesis. This review emphasizes the recent advances in the health benefits of probiotics and the emerging applications of probiotics in the food industry. Due to their capability to modulate gut microbiota and attenuate the immune system, probiotics could be used as an adjuvant in hypertension, hypercholesterolemia, cancer, and gastrointestinal diseases. Considering the functional properties, probiotics are being used in the dairy, beverage, and baking industries. After developing the latest techniques by researchers, probiotics can now survive within harsh processing conditions and withstand GI stresses quite effectively. Thus, the potential of probiotics can efficiently be utilized on a commercial scale in food processing industries.

## 1. Introduction

Probiotics, in the form of supplements or food products, have emerged as the most prominent ingredient in the era of functional foods. Probiotics have always been a vital component and commercial target for providing potential health benefits (Sanz et al., 2016; Hamad et al., 2022). The term “probiotic” was first presented by Werner Kollath in 1953, which is known to be a derivative of the Latin word *pro* and the Greek word *βιο* meaning “for life.” Kollath defined probiotics as active bodies with essential functions for promoting various health aspects (Gasbarrini et al., 2016). Food and Agriculture Organization (FAO) and World Health Organization (WHO) described them as “live microbes when administered in adequate quantities, confer health benefits on host organisms” (Munir et al., 2022). Several bacteria belonging to the genera *Pediococcus, Lactococcus, Enterococcus, Streptococcus, Propionibacterium*, and *Bacillus* are considered potential microbes for probiotic status (de Brito Alves et al., 2016; Hamad et al., 2022).

The frequently used strains belong to the divergent group of *Bifidobacterium* and *Lactobacillus* that significantly affect health with various actions. They detoxify xenobiotics and environmental pollutants (Reid, 2015), bio-transform mycotoxins in foods (Hamad et al., 2022), synthesize vitamin K, riboflavin, and folate (Reid, 2015; Hamad et al., 2022), and ferment undigested fiber in the colon (Warman et al., 2022). Probiotics prevent pathogenic bacteria by restricting binding sites on mucosal epithelial cells and modulating the host immune response, thus improving intestinal barrier integrity (Fusco et al., 2023). The advantages of probiotics are related to the modulation of gut microbiota, mitigation of nutritional intolerances (lactose intolerance), increase in bioavailability of macro and micronutrients, and alleviation of allergic incidences in susceptible individuals (Roobab et al., 2020).

Probiotics can be consumed either by incorporating them into foods or drinks in the form of dairy or non-dairy foodstuffs or as supplements (Fenster et al., 2019). Various fermented foods have active microbes genetically similar to the strains utilized as probiotics. It has been observed that fermented foods enhance the functional and nutritional aspects by transforming substrates and producing bioactive and bioavailable end-products (Marco et al., 2017). The approximate consumption of 10^9^ colony-forming unit (CFU)/day have been revealed as an effective dose (Hill et al., 2014). By keeping in view, the effective dosage, probiotics are being incorporated into many foods like beverages, ice cream, yogurt, bread, and many others by the food industry. The most significant barrier associated with probiotics in the food industry is their susceptibility to processing conditions and sensitivity to gastrointestinal (GI) stresses. However, regarding their health benefits, the consumer always showed an inclined interest in probiotic products (Konuray and Erginkaya, 2018). Now scientists have developed new and innovative methods like nanoencapsulation and genetic modification, which enable probiotics to withstand harsh conditions of both processing and GI stresses in the body (Putta et al., 2018). This review paper provides a profound insight into the mechanistic approach and current perspective on the beneficial aspects of probiotics in preventing and treating various diseases. The application and safe utilization of probiotics in major food industries have also been described.

## 2. Mechanisms of action

**Figure 1:**
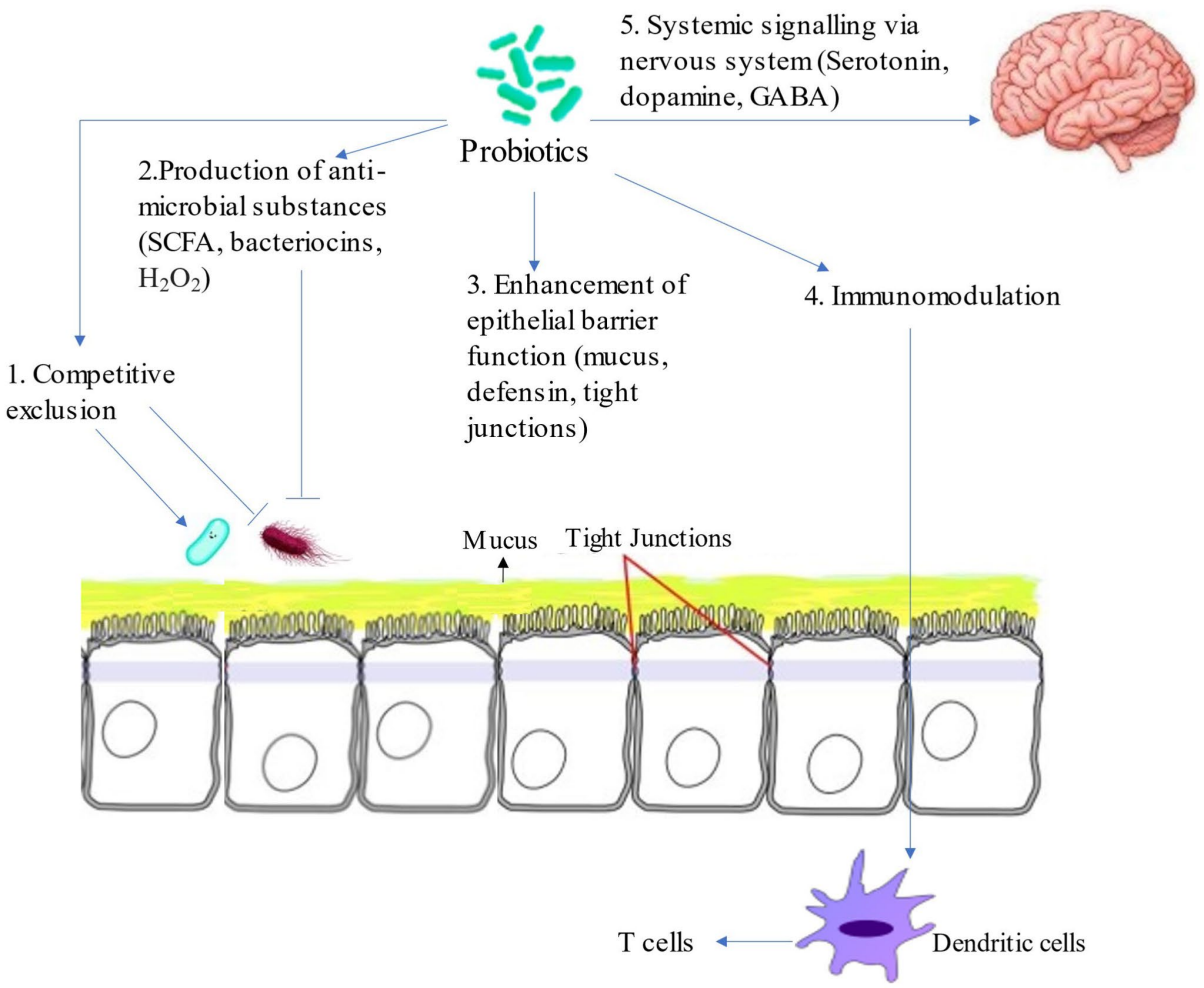
Mechanism of action of probiotics. 1. Probiotics perform their function by competing with pathogens for nutrients and receptors for binding thereby making their survival and adherence to gut mucosa difficult. 2. Probiotics produce anti-microbial substances which inhibit pathogens growth. 3. Probiotics promote epithelial barrier function by enhancing mucus production and increasing the expression of tight junction proteins which prevents the translocation of pathogens from intestine into the blood. 4. Probiotics regulate immunity of the host by modulating maturation and function of dendritic cells subsequently increasing the activity of T cells which play important role in immune homeostasis. 5. Probiotics also regulate the production of neurotransmitters including serotonin, dopamine and gamma aminobutyric acid (GABA).

Outstanding advances have been made in the field of probiotics, but there has yet to be a key breakthrough in the documentation of their mechanism of action. Probiotics possibly exert a positive potential on the human body through these main mechanisms; competitive exclusion of pathogens, improvement in intestinal barrier functions, immunomodulation in the host’s body, and production of neurotransmitters (Figure 1; Plaza-Diaz et al., 2019). Probiotics compete with pathogens for nutrients and receptor-binding sites, making their survival difficult in the gut (Plaza-Diaz et al., 2019). Probiotics also act as anti-microbial agents by producing substances; short chain fatty acids (SCFA), organic acids, hydrogen peroxide (Ahire et al., 2021), and bacteriocins (Fantinato et al., 2019) thus decreasing pathogenic bacteria in the gut. Moreover, probiotics improve the intestinal barrier function by stimulating the production of mucin proteins (Chang et al., 2021), regulating the expression of tight junction proteins, including occluding and claudin 1, and regulating the immune response in the gut (Bu et al., 2022; Ma et al., 2022).

Probiotics also regulate the innate and adaptive immune response modulating dendritic cells (DC), macrophages B and T lymphocytes. Probiotics also increase the production of anti-inflammatory cytokines while interacting with intestinal epithelial cells and attracting macrophages and mononuclear cells (Petruzziello et al., 2023). Furthermore, probiotics can produce neurotransmitters in the gut through the gut-brain axis. Specific probiotic stains can modulate the serotonin, gamma-aminobutyric acid (GABA), and dopamine levels, affecting mood, behavior, gut motility, and stress-related pathways (Srivastav et al., 2019; Sajedi et al., 2021; Gangaraju et al., 2022).

## 3. Health attributes of probiotics

**Figure 2:**
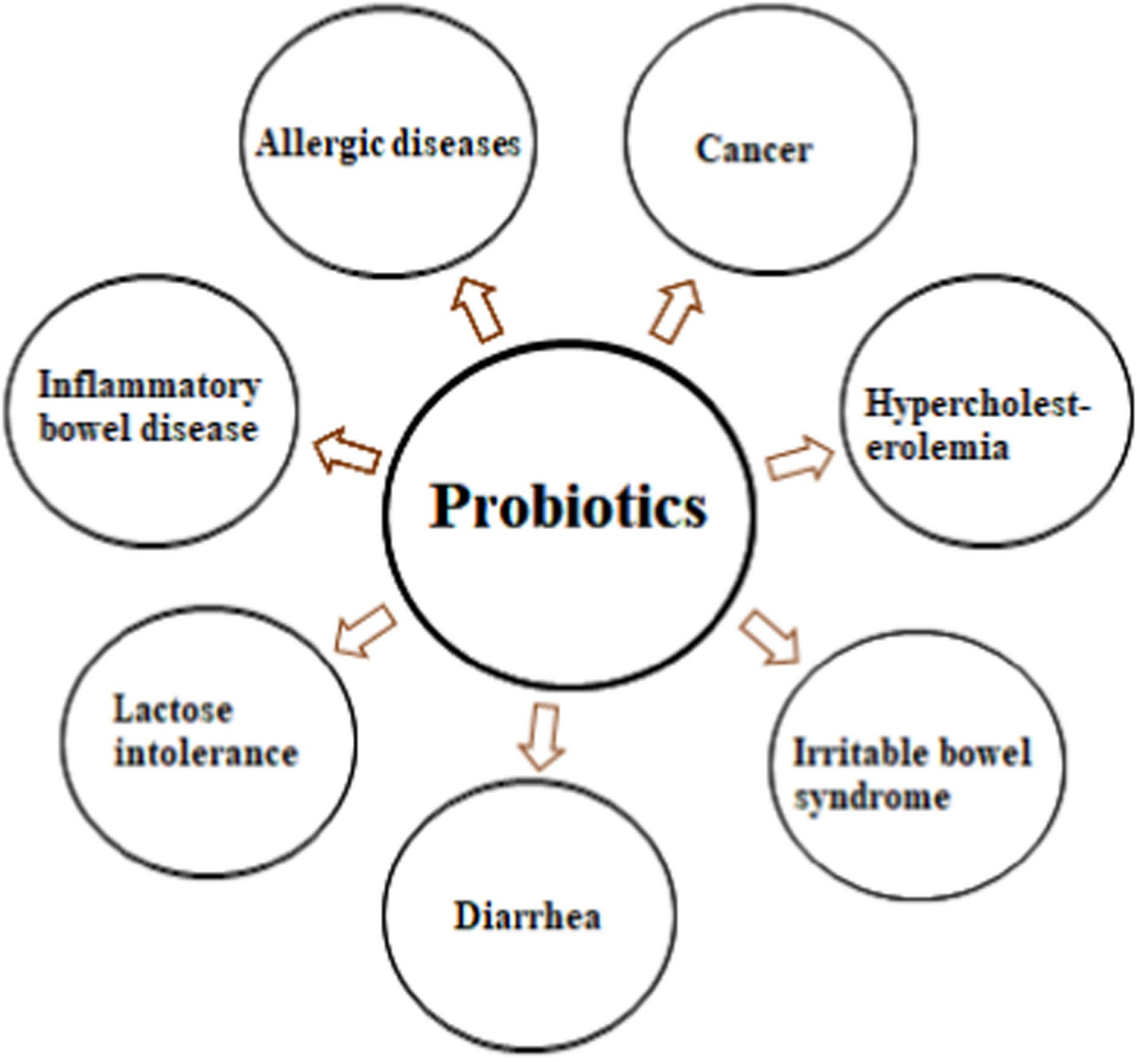
Health attributes of probiotics. Probiotics help in the prevention and management of allergic diseases, cancer, hypercholesterolemia, irritable bowel syndrome, diarrhea, lactose intolerance, inflammatory bowel disease.

The health benefits of probiotics are associated with preventing and reducing many diseases, i.e., allergic diseases, cancer, hypercholesterolemia, lactose intolerance, inflammatory bowel disease, diarrhea, and irritable bowel syndrome (Grom et al., 2020), as shown in Figure 2. Table 1 shows different studies regarding the application of probiotics in different diseases.

**Table 1:** Therapeutic effect of probiotics in gastrointestinal disorders.

Disease	Strain	Dosage	Subjects	Results	References
Allergic reactions	*L. plantarum*	5 × 10^10^ cells once a week for 4 weeks	Mice sensitized with peanut allergen	↓ Interleukin-10 ↑ Interferon-*γ*	Yang et al. (2021)
Allergic reactions	*Lactobacillus* multiple strains	10^9^ CFU lactobacilli every day for 28 days	30 BALB/c mice model of soybean sensitization	↑ Interferon-γ and IL-2 ↓ IL-4, IL-6 Promoted Tregs	Yang et al. (2021)
Cancer	*Lactobacillus fermentum*	–	CCD18-Co, HCT-116, and HT-29 cell lines	Activation of intrinsic apoptosis	Lee et al. (2019)
Cancer	*Pediococcus acidilactici* TMAB26	–	HT-29 and Caco-2 cell lines	Significant toxicity on cancer cells	Barigela and Bhukya (2021)
Hypercholesterolemia	*L. casei* pWQH01 *L. plantarum* AR113	1 × 10^9^ CFU for 5 weeks	30 male C57BL/6J mice	Have Bile Salt Hydrolase activity ↓ hepatic levels of TC and LDL-C ↑cholesterol 7α-hydroxylase (CYP7A1) gene	
Hypercholesterolemia	*L. fermentum* MJM60397	5 × 10^10^ CFU	Male mice	↓ cholesterol and low-density lipoprotein (LDL) cholesterol levels ↑ LDLR gene	Palaniyandi et al. (2020)
Ulcerative colitis	*Bifidobacterium longum* 536 (BB536)	2–3 × 10^11^ three times daily for 8 weeks	56 patients with mild to moderate UC	↓ Mayo subscore ↓Rachmilewitz endoscopic index (EI)	Tamaki et al. (2016)
Ulcerative colitis	*L. lactis* NCDO 2118	2.5 × 10^6^ CFU/g	36 mice	↓ Severity of colitis ↓ disease activity index ↑ gene expression of tight junction proteins (*zo-1*, *zo-2*)	Cordeiro et al. (2021)
Lactose intolerance	*L. acidophilus*	1 × 10^10^ once daily for 4 weeks	60 human participants	↓Abdominal pain ↓Abdominal cramping ↓Vomiting	Pakdaman et al. (2015)
IBS	*L. delbruekii* and *L. fermentum*	10 billion bacteria twice daily for 4 weeks	90 human subjects	↓Abdominal pain ↓IL-8 Restore normal intestinal flora	Husein et al. (2017)
Radiation-induced diarrhea	*L. acidophilus* and *B. animalis*	1.75 billion lyophilized live bacteria three times daily	53 patients receiving external beam pelvic radiotherapy	↓Moderate and severe diarrhea ↓Grade II abdominal pain	Linn et al. (2019)
Chronic diarrhea	*L. plantarum* CCFM1143	3.52 × 10^9^ CFU per day	55 human patients with chronic diarrhea	Improved clinical symptoms of diarrhea Improved immune response Modulated gut microbiota	Yang et al. (2021)
Antibiotic associated diarrhea	*Lactobacillus* and *Bifidobacterium* strains	1 × 10^9^ CFU once a day	36 human subjects	Delayed recurrence of diarrhea (5.39 days) ↓ Average no. of daily stools 45% positive evaluation	Trallero et al. (2019)
Chron’s disease	*B. longum* and inulin/oligofructose	2 × 10^11^ freeze-dried viable *B. longum* twice daily for 6 months	35 human subjects	↓Crohn disease activity indices ↓Histological scores ↓TNF-α expression	Steed et al. (2010)

↓ shows the reduction in different parameters while ↑ shows increasing trend.

### 3.1. Antiallergic effect of probiotics

Allergy is a hypersensitive disorder of the immune system, termed as type I hypersensitivity and defined as a “disease following a response by the immune system to an antigen.” With escalating incidence rate, allergies affect nearly half of the population of Europe and North America. These allergic reactions occur due to one or more common environmental substances or antigens (Prakash et al., 2014). The most common allergic reactions include asthma, rhinitis, atopic eczema, dermatitis, urticaria, angioedema, hay fever, and food, drug, and insect hypersensitivity (Lopez-Santamarina et al., 2021). The gut microbiome is a viable therapeutic target for managing allergic diseases (Harata et al., 2016), as they modulate the immunological and inflammatory response that consequently affects the development of sensitization and allergy (Fiocchi et al., 2015).

Allergic diseases are characterized by an imbalance in lymphocyte-governed immunity in which the immune response becomes overly biased toward T helper 2 lymphocytes dominated response (Th2 cells) (Di Costanzo et al., 2016). Allergen-sensitized Th2 cells produce various interleukins such as IL-1, IL-4, and IL-5, thus recruiting granular effector cells, i.e., mast cells, eosinophils, and basophils toward the site of allergic inflammation. In addition, the interleukins switch B lymphocyte immunoglobulin isotype, which upsurges the circulating level of total and allergen-specific IgE (Galli et al., 2020). Although the precise mechanism is not entirely known, it is expected that the probiotics improve mucosal barrier functions, stimulate the immune system, reduce leakage of antigen through the mucosa, produce anti-inflammatory cytokines, increase the production of secretory IgA (exclude antigens from intestinal mucosa), degrade dietary antigen and up-regulate anti-inflammatory cytokines as IL-10 (Liang et al., 2022).

**Figure 3:**
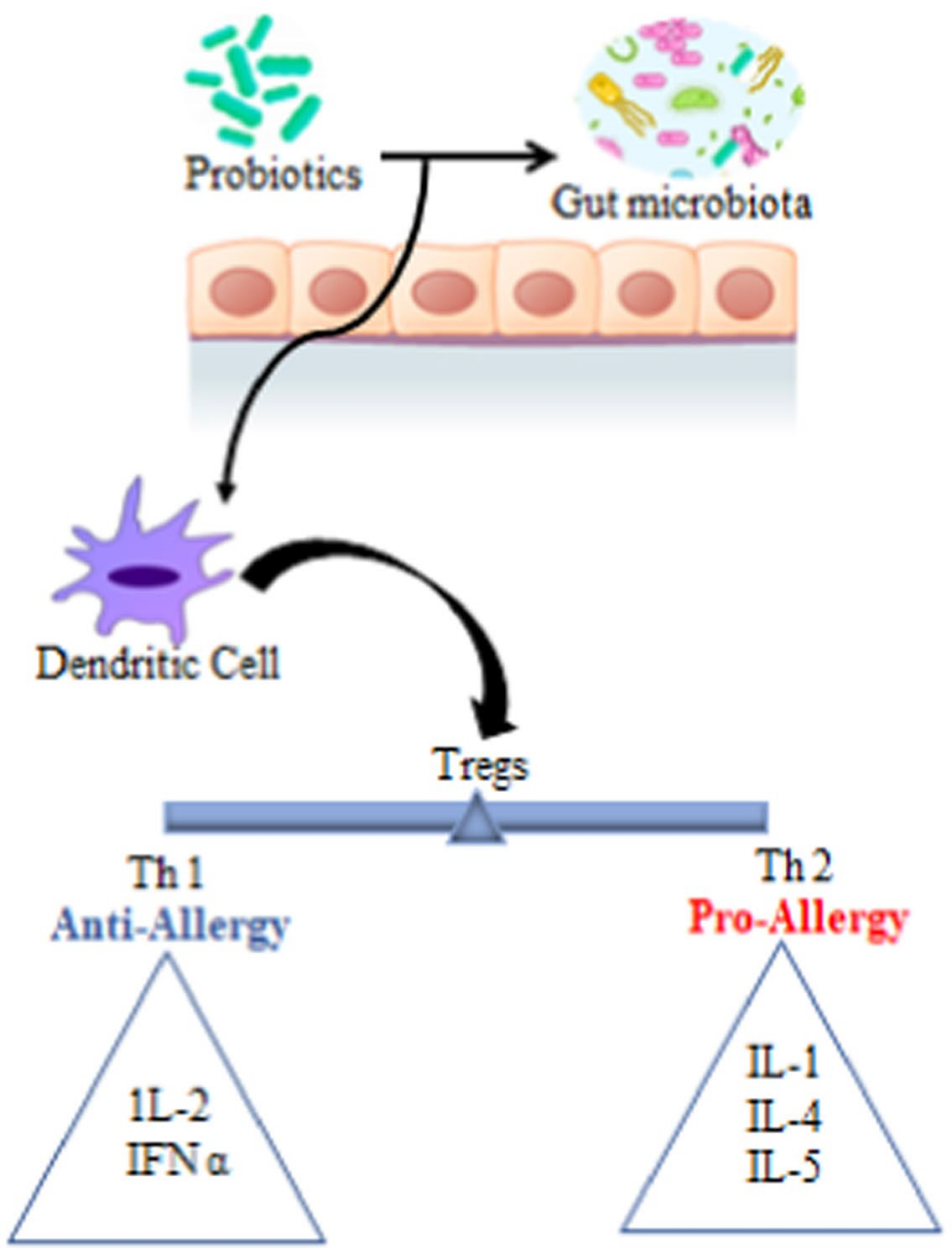
Anti-allergic effect of probiotics. Tregs, T regulatory cells; Th 1, T helper cells type 1; Th 2, T helper cell type 2; IL, interleukin; IFN α, interferon α. Probiotics help in the migration and maturation of dendritic cells via modulating the composition of gut microbiota. Dendritic cells in the gut-associated lymphoid tissues have the ability to induce the development of peripheral Tregs and to play a central role in the development of immune homeostasis. Tregs maintain the proper level of Th 1, Th 2 cells as well as anti-allergy and pro-allergy cytokines.

The proposed mechanism for the antiallergic effect of probiotics is the augmentation of T helper cells (Th)1/Th2 immune balance by suppressing Th2 skewed immune response and favoring Th1 cell response (Di Costanzo et al., 2016). Ma et al. (2019) explain that probiotics modulate the function of dendritic cells, which in turn have the ability peripheral Tregs. Tregs control the excess immune response and maintain a balance between Th1 and Th2 cells (Figure 3). Besides, lactobacilli stimulate regulatory T cells which play a paramount role in balancing immune response through the production of immunosuppressive cytokines and modulation of IgE, IgA, and IgG production (Owaga et al., 2014).

The antiallergic effect of *Lactiplantibacillus plantarum* SY12 and *L. plantarum* SY11 was studied using RAW 264.7 (murine macrophage) cell line. Both species showed a reduction in the production of nitric oxide, T helper 2 linked cytokines, tumor necrosis factor-α, and cyclooxygenase-2 as well as inducible nitric oxide synthase compared to the control group (Lee et al., 2014). In this regard, the *Limosilactobacillus reuteri* effect was also investigated against the food allergy in ovalbumin (OVA)-sensitized BALB/c mice. Oral intake of *L. reuteri* helped restore the deteriorated profile of colonic microflora and attenuated allergic diarrhea. It also increased the activation of mast cells, enhanced the production of serum immunoglobulin E (IgE), suppressed the T helper 1 and 2 cytokines production, down-regulated the GATA3 expression, and increased the expression of TGF-b, IL-10, and Foxp3. The findings confirmed the anti-allergic activities of *L. reuteri* promoted by the modulation of enteric flora and enhancement of tolerogenic immune responses (Huang et al., 2017).

### 3.2. Cancer suppressor activity of probiotics

**Figure 4:**
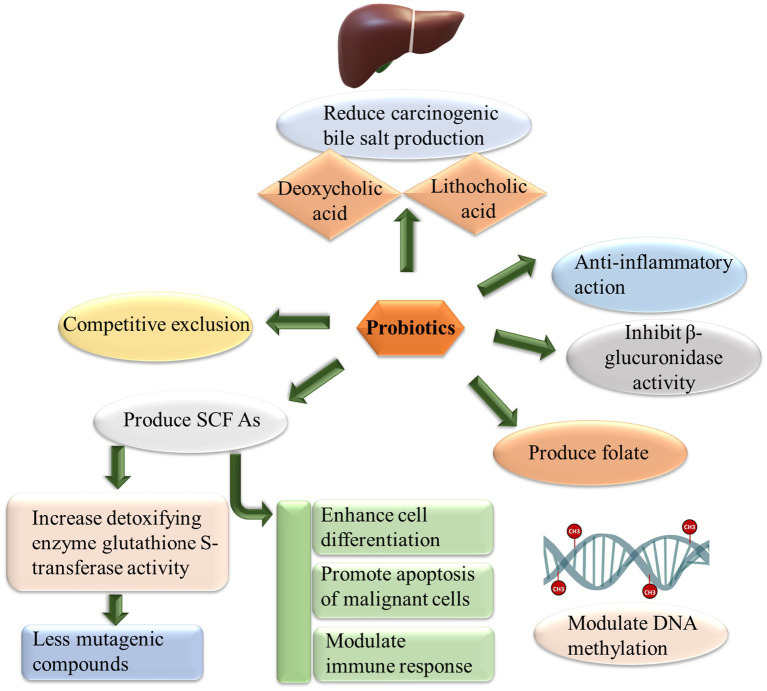
Cancer suppressor activity of probiotics. Probiotics use different pathways to fight against cancer. Probiotics inhibit β glucuronidase activity, produce folate which ultimately modulate DNA methylation patterns protecting the integrity of genome, produce short chain fatty acids (SCFA) enhancing cell differentiation and apoptosis of cancerous cells, exclude pathogens involved in chronic inflammation which may lead to cancer development.

Probiotics could be used as an adjuvant for various types of cancers based on their potential to modulate enteric flora and enhance local and systematic immunity. They prevent the initiation, progression, and metastasis of transplantable or chemically induced tumors (Samanta, 2022). The effect of probiotics can be observed in suppressing both intestinal and extraintestinal cancers (So et al., 2017). The interaction of probiotics and their metabolites (bacteriocin, peptides, and organic acids) with critical metabolic pathways such as cellular proliferation, inflammation, apoptosis, angiogenesis, and metastasis has been revealed by many researchers (Harikumar et al., 2013). Moreover, the probiotics inhibit carcinogenesis by inhibiting pathogens through competitive exclusion, increasing short-chain fatty acid production (Chong, 2014), reducing carcinogenic bile salts production, binding carcinogens and mutagens, down-regulating NF-kappa B dependent genes products for cell proliferation (Cox-2, cyclin D1) and cell survivability (Bcl-3, Bcl-xL) and enhancing apoptosis (Konishi et al., 2016). Probiotics also upregulate TNF-related apoptosis-inducing ligand (TRAIL) (Klłonowska-Olejnik, 2004), modulate cell cycle by rapamycin (mTOR)/4EBP1 (Islam et al., 2014) and inhibit the formation of aberrant crypt foci (Yu and Li, 2016). Figure 4 describes the anti-cancer effect of probiotics.

Previous studies have scrutinized that the ERK1/2 pathway modulates cell survival, proliferation, differentiation, and cell motility by regulating the BCL-2 protein family in mitochondria (Passaniti et al., 2022). *Saccharomyces boulardii,* both *in vitro* and *in vivo*, inhibited the activation of ERK1/2 mitogen-associated protein kinase. In the same way, probiotic *L. reuteri* induced apoptosis in human myeloid leukemia-derived cells by modulating NF-kappa B and MAPK signaling pathways (Saber et al., 2017). The colonic microflora has also been related to the development of liver disorders such as liver fibrosis (De Minicis et al., 2014), nonalcoholic fatty liver diseases (Zhuge et al., 2022), and more recently, liver cancer (So et al., 2017). Probiotics have been demonstrated to inhibit hepatocellular carcinoma (HCC) progression by reducing liver tumor size and down-regulating angiogenic factors. The mechanistic approach to this is the level of T helper (Th) 17 cells in the gut and its recruitment to tumor sites was lower in probiotic-treated mice (Li et al., 2016). In breast cancer apart from immunomodulation, the hypoxia-inducible factor (HIF) pathway was also reported to be significantly suppressed by *Lactobacillus* cultures supernatant (Esfandiary et al., 2016).

In addition to this, experimental studies were carried out to reduce the mutagenic potential of a powerful carcinogen; *N*-methyl-*N*′-nitro-*N*-nitrosoguanidine (MNNG) by *Lacticaseibacillus rhamnosus* Vc. Oral feeding of *L. rhamnosus* Vc (10^9^ CFU) to *Gallus gallus* (chicks) for 30 days significantly detoxified the parent compound reducing its mutagenicity (61%) and genotoxicity (69%) (Pithva et al., 2015). In another study, the role of *Saccharomyces cerevisiae* on the activation of apoptotic pathway Akt/NF-kB was explored in cancer. Heat-killed *S. cerevisiae* induced apoptosis in cancer cells, the SW480 cell line, by up-regulating Bax, cleaved caspase 3 and cleaved caspase 9, and down-regulating p-Akt1, Bcl-XL, Rel A, procaspase 3 and procaspase 9 expressions. Hence, it was concluded that probiotics modulate Akt/NF-kB pathway following the apoptotic cascade and play an essential role in cancer prevention (Shamekhi et al., 2020).

### 3.3. Hypocholesterolemic effect of probiotics

**Figure 5:**
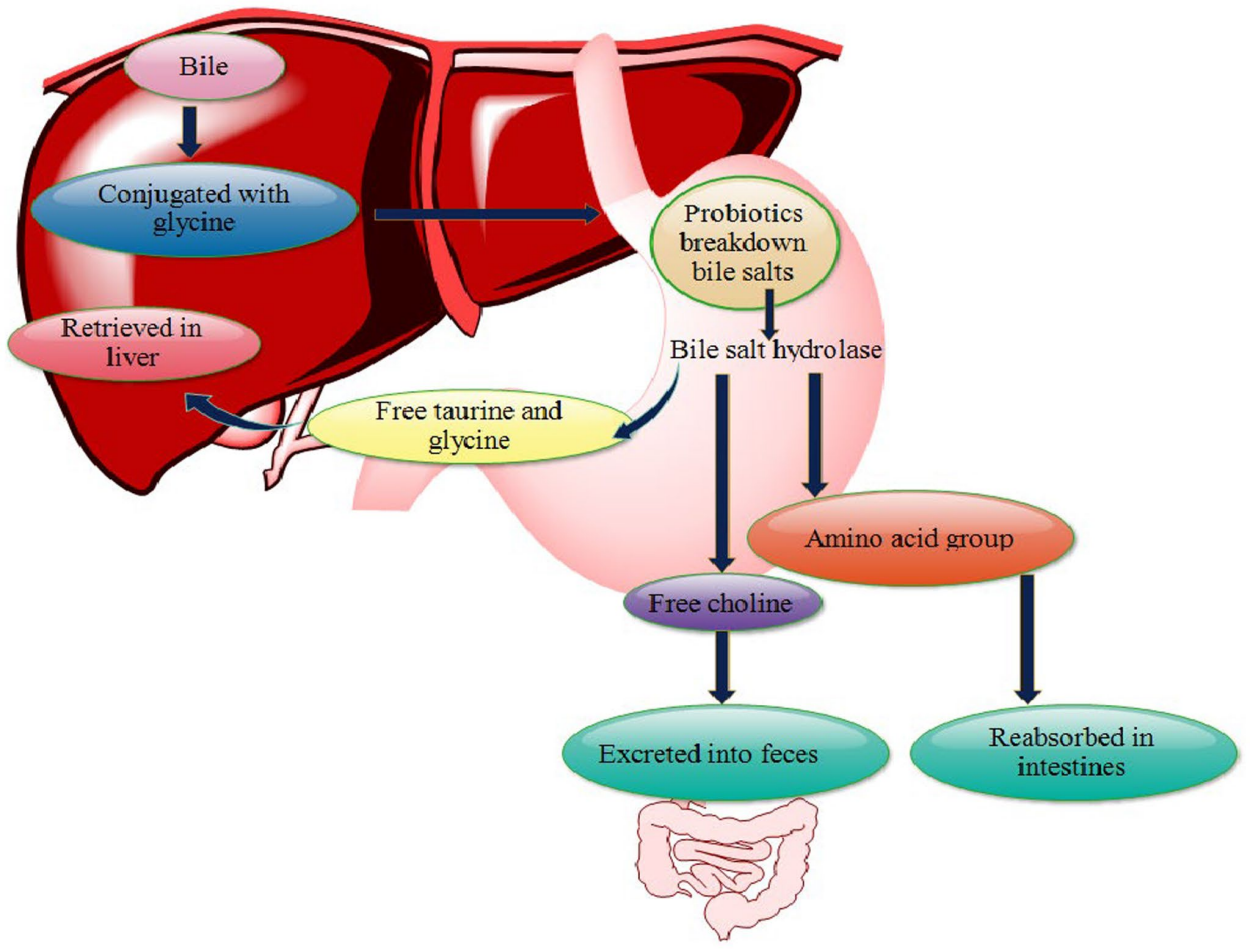
Mechanism of lowering cholesterol level by probiotics. Probiotics breakdown or deconjugate bile salts into free choline, glycine and amino group by synthesizing bile salt hydrolase. Free choline excreted via choline, amino acid group is absorbed in the intestine, and free taurine and glycine return back to the liver. This increases the elimination of bile from body and more cholesterol is used to synthesize bile thereby, reducing the cholesterol level in the blood.

Probiotics can be used as an effective tool for lowering blood cholesterol levels. They can act directly or indirectly to decrease cholesterol levels in the body. The direct mechanism includes the inhibition of *de novo* synthesis of cholesterol by hypocholesterolemia factors like uric acid, lactose, orotic acid, and whey protein as well as the reduction in intestinal absorption of dietary cholesterol in three ways- assimilation, binding, and degradation (Thakkar et al., 2016). The indirect mechanism for curtailing cholesterol by probiotics is deconjugating bile salts (conjugated glycodeoxycholic acid and taurodeoxycholic acid) via bile salt hydrolase (BSH) production. Deconjugated bile salts are less reabsorbed through the intestine, thus inhibiting enterohepatic circulation of the bile and higher excretion in the feces (Figure 5; Rezaei et al., 2017).

Human and animal studies have provided evidence for the hypocholesterolemic properties of probiotics. In a study, the hypocholesterolemic properties of *Levilactobacillus brevis* MT950194 and *L. brevis* MW365351 were observed both *in vitro* and *in vivo.* The strains reduced cholesterol content, increased fecal cholesterol excretion, and converted bile into free cholic acid (Munir et al., 2022). The potential of a probiotic complex comprising *Pediococcus*, *Lactobacillus*, and *Bifidobacteria* was also investigated in lipid metabolism. After 10 weeks of the experimental period, the results showed significantly reduced cholesterol levels in medium and high-dose groups (Galli et al., 2020). The cholesterol reduction potential of a new strain, *L. plantarum* DMDL 9010, was investigated by using *in vivo* model. The intake of strain resulted in the reduction of serum cholesterol, hepatic cholesterol, triglycerides, and an increase in fecal excretion of bile acids. A significant decrease in total cholesterol, low-density lipoprotein, and atherosclerosis index by 23.03, 28.00, and 34.03%, respectively was observed with the use of *L. plantarum* DMDL 9010 (10^9^ cells per day) (Liu et al., 2017).

Recently, research regarding gene expression by probiotics in hypercholesterolemia was conducted by Dehkohneh and his colleagues. The role of *Lacticaseibacillus paracasei* TD3 was examined in modulating two significant genes involved in cholesterol metabolism; 3-hydroxy-3-methyl glutaryl coenzyme (HMGCR) and cytochrome P450 7A1 (CYP7A1). A dose of 1 × 10^10^ CFU was given to male Wistar rats for 21 days. The cholesterol level was significantly decreased along with the reduction of alanine aminotransferase (ALT) and aspartate aminotransferase (AST) enzymes. The dramatic decline of HMGCR and CYP7A1 genes in adipose tissues was also observed using real-time polymerase chain reaction (Dehkohneh et al., 2019).

### 3.4. Impact of probiotics on intestinal diseases

The gut plays a pivotal role in the digestion and absorption of nutrients and maintains mucosal barrier integrity. Numerous commensal bacteria reside in the human GI tract constituting an active community, which strongly affects human physiology (Shehata et al., 2022). The modification in intestinal microflora can be achieved by administering antibiotics, probiotics, prebiotics, and fecal transplant (Shahverdi, 2016).

The metabolic activity of the intestinal microbiome affects the host’s health, both favorably and unfavorably (Saber et al., 2017). The exact balance in the microflora (eubiosis), when disturbed, results in acute and chronic clinical disorders like antibiotic-associated diarrhea (AAD), ulcers, inflammatory bowel disease (IBD), and irritable bowel syndrome (IBS) (Saber et al., 2017). In addition, several researchers have supported the theory that microbial dysbiosis participates in the etiology of some human cancers (Su et al., 2021), especially GI cancers (Pereira-Marques et al., 2019). Restoring healthy gut microbiota can be used as a practical approach to managing intestinal diseases. Probiotics can increase microbial richness and diversity, increase enzyme (Lactase) production, improve immune micro-environment (Jang et al., 2019), and improves intestinal permeability (Stratiki et al., 2007). In this way, probiotics can alleviate intestinal diseases. Studies regarding the use of probiotics in intestinal diseases are given in Table 1.

## 4. Application of probiotics in the food industry

The public awareness of diet-related issues and ever-increasing evidence about probiotic health benefits have increased consumer interest in probiotic foods. A large number of food items, including yogurt, powdered milk, frozen fermented dairy desserts, cheese and cheese products, ice creams, baby foods, cereals, and fruit juices, are among numerous probiotic foods (Papademas and Kotsaki, 2019). The most prominent barrier to using probiotics in the food industry is their sensitivity toward heat treatments during processing and GI stresses in the human body. However, researchers and food industries are trying to find new and innovative methods and techniques to overcome the issues (Zhang et al., 2022). The global increase in sales of probiotics-based products is estimated to reach 75 billion dollars by 2025. This exponential growth in sales of probiotic products has already gained much interest from food producers to develop new products with probiotics. Probiotics are commonly used in dairy, beverage, baking, and edible film industries (Reque and Brandelli, 2021).

### 4.1. Probiotics in the dairy industry

Food producers have been showing great interest in developing new probiotics products due to their large acceptability among consumers. Dairy-based products are prepared as natural products to promote health and prevent diseases (Nami et al., 2019). Lactic acid bacteria (LAB) in dairy products help increase the shelf life of fermented products. LAB act as antimicrobial agents against many pathogens living inside the human body, thus improving human health (de Souza da Motta et al., 2022). Table 2 refers to the application of probiotics in the dairy industry. Considering the demand for functional dairy products in markets, it has been estimated and forecasted that the industry will jump up to a market value of 64.3 billion USD globally by the end of 2023, apart from traditional dairy products (Iqbal et al., 2017; FAO, 2022).

**Table 2:** Application of probiotics in food industries.

Food industry	Product	Probiotic strain	Storage time	Viability at the end of storage	References
Dairy	Ricotta cheese	*B. animalis* subsp. *lactis* (Bb-12) *L. acidophilus* (La-05)	7 days at 7°C	∼10^6^ CFU/g	Meira et al. (2015)
	Yogurt	*B. Lactis*	29 days at 4°C	10^6^–10^7^ CFU/g	Danielle (2015)
		*L. acidophilus B. animalis* subsp. *lactis*	45 days at 5 ± 1°C	8.84 log CFU/g 8.01 log CFU/g	Lucatto et al. (2020)
	Cheddar cheese	*L. lactis* subsp*. lactis L. helvetics S. thermophilus L. rhamnosus*	4 weeks at 16°C	10^8^ CFU/g	Ulpathakumbura et al. (2016)
	Mango juice enriched dairy drink	*L. acidophilus*	5 weeks at 4 °C	7.72 log CFU/mL	Leaf et al. (2016)
Beverages-fruit based	Pineapple juice	*L. acidophilus, L. plantarum*, and *L. lactis*	60 days at 4°C	9–10 log CFU/mL	Nguyen et al. (2019)
	Orange juice	*P. acidilactici*	35 days at 4°C and 30°C	7.2–8.5 log CFU/mL	Cristiny de Oliveira Vieira et al. (2020)
	Pomegranate	*L. plantarum* ATCC 14917	28 days at 4°C	8.8 log CFU/mL	Mantzourani et al. (2018a)
	Cornelian cherry juice	*L. plantarum*	4 weeks at 4°C	9.95 log CFU/mL	Mantzourani et al. (2018b)
Beverages-vegetable based	Carrot blended with orange juice	*L. plantarum* CECT 220	30 days at 4°C	10^8^–10^9^ CFU/mL	Al-Sheraji et al. (2013)
	Beet	*L. plantarum*	21 days at 4°C	7–8 log CFU/mL	Barbu et al. (2020)
	Melon, carrot	*L. plantarum* CICC22696 and *L. acidophilus* CICC20710	28 days at 4°C	10^8^–10^9^ CFU/mL	Do and Fan (2019)
Bakery	Pan bread	Sodium alginate and 2% whey protein concentrate *L. rhamnosus* GG	7 days at room temperature	7.57–8.98 and 6.55–6.91 log CFU/portion	Lu et al. (2018)
	Bread	Encapsulating *L. acidophilus* and *L. casei* in calcium alginate	4 days at ambient temperature	7.2 × 10^8^ CFU/g	Seyedain-Ardabili et al. (2016)

Many products, such as pasteurized milk, infant formula, fermented milk, and ice creams are being produced and consumed worldwide as probiotic-based dairy products. Some products like cheese and fermented milk are preferred as probiotics carriers because their pH buffering capacity and fat contents give additional protection to probiotics while passing through the GI tract (Meybodi and Mortazavian, 2017). Yogurt, including reduced lactose or lactose-free, functional ingredient-supplemented yogurts such as vitamins, minerals, sterols, stanols, conjugated linoleic acids, prebiotics, and probiotics have also gained good market success for quite a long period (Fernandez and Marette, 2017).

Nowadays, probiotics-based dairy products have been recommended as safe and healthy due to their beneficial effects on health, such as aiding mineral absorptions in the body, being efficient against *Helicobacter pylori* infection, and preventing diarrhea and constipation (Gao et al., 2021). Nami and his team (Nami et al., 2019) found the hypocholesterolemic effects of *L. plantarum* from homemade yogurt. They found the most substantial cholesterol-removing potential in growing cells (84%), moderate removal of cholesterol in the resting cell (41.1%), and the lowest in dead cells (32.7%). *L. plantarum* showed a positive potential for controlling serum cholesterol. At the same time, it was found that *L. plantarum* was resistant to BSH activity, antibiotics, and hemolytic activity (Nami et al., 2019). Lee et al. (2020) prepared *L. plantarum* B710 containing fermented milk, which showed bone-protective effects. Moreover, Prezzi et al. (2020) examined that the addition of *L. rhamnosus* inhibited the growth of *Listeria monocytogenes* in Minas Frescal cheese*. L. rhamnosus* showed no negative effect on the textural and physiochemical properties of cheese and survived during storage and after simulated gastrointestinal conditions.

Arbex et al. (2018) investigated six *Leuconostoc mesenteroides* strains from three different sources of dairy and non-dairy products provided each sample showing probiotic properties. One strain of *L. mesenteroids* from camel milk coded as CM9 showed high dextran production and the best resistance to intestinal stresses. CM9 had a strong antimicrobial potential against *Staphylococcus aureus* and *Escherichia coli* (Arbex et al., 2018; Azam et al., 2021). In another research, the effect of *Lactobacillus acidophilus* and *L. rhamnosus* were investigated on soft cheese. It was found that *L. acidophilus* had good overall quality with a better immune-modulation response in mice. At the same time, they also controlled pro-inflammatory cytokines and interleukin regulation and enhanced the secretion of secretory immunoglobulin A (Cuffia et al., 2019). In a study, Nguyen et al. (2019) and Riaz et al. (2019) investigated the survival of *Bifidobacterium bifidum* encapsulated in zein. The results suggested that probiotic bacteria survived well after 32 days of storage (Nguyen et al., 2019).

### 4.2. Probiotics in the beverage industry

The demand for non-dairy probiotic foods has been increasing steadily, especially when the consumer has become aware of the side effects associated with medicine. Consuming probiotic food is more readily acceptable to consumers as it is a more natural way of receiving their daily dose of probiotics (Reque and Brandelli, 2021). Fruit juices supplemented with probiotics have been reported as a more unique and appropriate method in the probiotic beverage industry. Fruit juices have been accepted widely among all consumers regardless of age, gender, and geographic region around the globe due to the presence of essential nutrients (Mantzourani et al., 2018a,b). The viability of probiotics is shorter in non-dairy foods when compared to dietary supplements due to the harsh environments faced by probiotics in beverages. Processors must consider many factors in the production of probiotic juices, such as pH, temperature, anthocyanins, and most importantly a vegetative form of probiotics (Min et al., 2019; Azam et al., 2022).

To overcome these complexities, microencapsulation techniques have been introduced. Using these techniques, probiotics can be employed as an essential ingredient in the functional food industry. The micro or nanoencapsulation of probiotics allows them to withstand harsh processing and storage environments due to the protective coating around them (Afzaal et al., 2022). It was reported that the acid sensitivity of *Bifidobacterium* and *Lactobacillus* was improved after their microencapsulation with gelatin or plant gums (Ozturk et al., 2021). Besides this, low-temperature processing is also an effective strategy to control metabolic activity and protect probiotic cell viability throughout the shelf life of juices so that an adequate and safe dose of microbes is delivered to the consumer (Tyutkov et al., 2022). Some studies regarding probiotics in the beverage industry are shown in Table 2.

Miranda et al. (2019) have investigated the direct addition of an activated and microencapsulated form of probiotics in orange juice to check their effect on physical, chemical, rheological, microbial, and sensory parameters. They found that in the inactivated state, the level of organic acids was increased, but the essential volatile compounds were decreased. On the other hand, the encapsulated probiotics showed improved consistency and rheological parameters but their sensory attributes were not up to the mark due to changes in taste. The most optimum treatment was found to be the direct addition of probiotics to juice based on good physicochemical and sensory acceptance that was more similar to the natural pure product having many essential volatile compounds (octanol, o-cymene, α-cubebene, and 1-hexanol, etc.) (Miranda et al., 2019). Secondary packaging is another important technique used to produce shelf-stable beverage products. In this technique, the probiotics are in a separate compartment from food, i.e., bottle cap or straw, and are released only into juices immediately before consumption (Fenster et al., 2019).

In another research, water kefir grains were used to ferment soy whey (a byproduct of tofu) to prepare a functional beverage. After 2 days of fermentation, the polyphenol contents and antioxidant properties increased significantly, supported by good sensory scores and overall acceptability (Fenster et al., 2019). Laali et al. (2018) used *L. plantarum* to make a beverage from coconut water after fermentation. This process not only enhanced the vitamin and mineral (potassium, calcium, and sodium) contents but also improved anti-hypertensive, antioxidant, and antimicrobial properties making it suitable for use (Laali et al., 2018). The beverage prepared from whey, germinated millet flour, and barley extract was treated with *L. acidophilus* in another study, and it was found to be effective in controlling the pathogenicity induced by *Shigella* in mice models. The beverage stimulated the immune response and enhanced the IgA level, thus controlling pathogenicity (Ganguly et al., 2019).

### 4.3. Probiotics in bakery

Bakery products (bread, biscuits, doughnuts, cookies, etc.) contribute to several major food components such as carbohydrates, proteins, fats, dietary fiber, vitamins, and minerals in varying amounts (Niesche and Haase, 2012; El-Sohaimy et al., 2019). Researchers have been trying to incorporate probiotics in baked products by developing new techniques to deliver thermo-durable bioactive materials so that probiotics can survive high temperatures during baking (Mirzamani et al., 2021).

The microencapsulation technique and the sourdough method have been studied as an alternative to increasing the nutritional value and cell viability of probiotics in bread during baking (Ganguly et al., 2019) and in GI conditions (Champagne et al., 2018; Ashraf et al., 2022). In a study, *L. rhamnosus* was encapsulated in sodium alginate, and higher cell viability was observed during the baking of pan bread and in simulated gastrointestinal conditions (Hauser and Matthes, 2017). Zhang et al. (2018) analyzed the encapsulation of *L. plantarum* into bread-making using different matrices (reconstituted skim milk, gum arabic, maltodextrin, and inulin). The results suggested that bacterial survival was better in gum arabic and reconstituted skim milk than in the other two heating methods (Zhang et al., 2018). Another research studied the incorporation of *L. plantarum* under different baking temperatures (175, 205, and 235°C) and its survival during storage. The bacterial cell viability was counted every 2 min during baking and a decline from 10^9^ CFU/g to 10^4–5^ CFU/g was observed after baking. The storage results were remarkable as the probiotic viability was increased by 2–3 logarithmic cycles to 10^8^, which was attributed to the decline in the pH of bread during storage (Zhang et al., 2018). Table 2 illustrates the use of probiotics using different strains in the baking industry.

### 4.4. Probiotics in edible food coatings

Bioactive food packaging is the latest approach promoting the concept of functional foods due to its extraordinary health-promoting benefits. This technique is quite helpful in overcoming the stability and GIT stresses faced by probiotics (Khodaei and Hamidi-Esfahani, 2019). Studies on the use of probiotics with some biopolymers for edible coating are illustrated in Table 3.

**Table 3:** Use of probiotics in edible film.

Application matrix	Probiotic	Biopolymer material	Viability	References
Baked cereal products	*L. rhamnosus* GG	Sodium alginate	7.57–8.98 log CFU/portion	Lu et al. (2018)
	*L. acidophilus L. rhamnosus*	Carboxymethylcellulose (CMC)	10^7^ CFU/g	Ebrahimi et al. (2018)
Hake fillets	*B. animalis* spp. *lactis*, *L. paracasei* spp. *paracasei*	Agar	–	De Lacey et al. (2014)
	*L. rhamnosus* GG	Sodium alginate/Pectin/κ-Carrageenan-Locust bean gum/Gelatine/Whey protein concentrate	0.87–3.06 log CFU/g	Soukoulis et al. (2017)
	*L. reuteri* ATCC 55730 *L. rhamnosus* GG ATCC 53103 *L. acidophilus* DSM 20079	Pullulan and starches (from potato, tapioca, and corn)	12.9 log CFU/mL	Kanmani and Lim (2013)

The encapsulation of probiotics into edible films protects them from premature degradation and increases their viability in the human body (Singh et al., 2019). The technique of edible films is being used nowadays as a tool for the effective delivery of probiotics to consumers. Still, at the same time, it also enhances the stability and safety of food by inhibiting the growth of spoilage microorganisms (Pavli et al., 2018). The prime difference between active packaging and edible coating or bioactive packaging is that active packaging is usually done to enhance the safety and quality of packaged food, while on the other hand, bioactive packaging affects the health of consumers directly generating healthier packaged foods through edible coated bioactive material which upon consumption promote health (Gagliarini et al., 2019).

Many researchers have shown keen interest in film-forming materials, for instance, biopolymers including cellulose, zein, seaweed extracts, pectins, alginates, and chitosan for entrapping probiotics to enhance the nutritional values of foods (Pop et al., 2019). Therefore, bacterial microorganisms are being incorporated into films and coatings to confer probiotics’ ability to the food products or act as antimicrobial agents (Afsah-Hejri et al., 2013). As an example, the fabricated cellulose-based edible films in combination with *L. rhamnosus* using sodium carboxymethyl cellulose (CMC) and hydroxymethyl cellulose (HEC) with citric acid as a crosslinker to control the consistency of film loaded with *L. rhamnosus* (Singh et al., 2019). Moreover, cellulose-based edible films showed the therapeutic effects of probiotics (Singh et al., 2019). The film effect provides a suitable environment to encapsulate bacteria from transport to delivery in the GIT system effectively.

Four probiotic strains (*L. acidophilus*, *L. casei*, *L. rhamnosus*, and *B. bifidum*) were investigated using CMC-based edible coatings in this regard and their effects on storage under refrigerated conditions were also checked. The results suggested that *L. acidophilus* showed the highest viable count during storage with more water vapor permeability and opacity and decreased tensile strength and elongation at break values of film structure. The physical and mechanical properties of edible films remained the same (Ebrahimi et al., 2018). Another research found that after incorporating *L. plantarum* into CMC-based edible coating, the physicochemical properties and microbial characteristics of fresh strawberries were significantly improved. The probiotics population remained constant throughout the storage period, which controlled mold and yeast growth and helped to improve the shelf life of strawberries (Khodaei and Hamidi-Esfahani, 2019).

Bambace et al. (2019) incorporated *L. rhamnosus* into an alginate prebiotic fiber solution to enhance the shelf life of minimally processed and ready-to-eat blueberries by fourteen days. *L. rhamnosus* showed good antimicrobial properties with alginate and sensory acceptability for coated food (Bambace et al., 2019). In another work, kefiran polysaccharides-based films were used to deliver probiotics (*L. paracasei* and *Kluyveromyces marxianus*) to the gut. These films exhibited good antimicrobial properties and protected the probiotics from GIT stresses. *L. paracasei* showed better mechanical properties and good viable count than *K. marxianus* (Gagliarini et al., 2019).

## 5. Delivery systems and the strategies to extend viability

The association between probiotics and human health has been well-known for an extended period. When consumed orally, probiotics can regulate the composition of intestinal microbiota (Sharma et al., 2023). However, the severe physicochemical stresses (high temperatures and acidity during processing, storage, and passage to the large intestine) can drastically reduce the viability of probiotics. Researchers have used different encapsulating techniques to overcome these stresses and enhance the viability of probiotics within the human body (Luo et al., 2022). The traditional and most widely used technique is microencapsulation. Microencapsulation is classified into four methods, namely; spray drying, freeze drying, emulsification, and extrusion. One can improve the ability of probiotics to withstand the harsh environment of processing and the human body. Still, these methods have certain limitations, such as extreme temperatures and acidity can ultimately affect the size, stability, and ultimately viability of microstructures of microcapsules (Razavi et al., 2021).

These hindrances paved the way to find new encapsulation strategies to enhance the durability and viability of probiotics. In recent years, the nanoencapsulation technique has been used widely to enhance probiotics-loaded nanoparticles’ ability to face severe processing and *in-vivo* stresses. These techniques also facilitate the targeted delivery and control release of probiotics in the intestine (Xu et al., 2022). The unique biological and physicochemical characteristics of nanocapsules, such as smaller particle sizes, higher surface areas, and increased reactivities, improve the efficiency of encapsulated probiotics, thus, providing a logical solution to human health and safety (Singh et al., 2022). The ability of nanoencapsulation to entrap probiotics is analyzed by the potential of electrospun nanofibers, hydrogels, nanocoating, nanoliposomes, and other nanomaterials (Garcia-Brand et al., 2022).

Mojaveri and his colleagues, in their recent work, attempted to improve the viability of *Bifidobacterium animalis* Bb12 by using a nanofiber technique made from chitosan and poly (vinyl alcohol) and inulin as prebiotics. The simulated results of the GI tract showed that the encapsulation of probiotics in electrospun nanofibers significantly enhanced the physicochemical behavior with increased stability of nanoparticles within the human body (Mojaveri et al., 2020). In another study, Li et al. (2019) studied the cellulose-based gels for control release of encapsulated *L. plantarum* with better storage and concluded that cellulose-based gels provide better storage stability and much-enhanced control release pattern in simulated intestinal fluids (Li et al., 2019).

Encapsulation of probiotics with the help of biomaterial-based nanocoating can also protect these beneficial microbes from antibiotics and GI conditions, facilitating the retention of probiotics within the GI tract. It was found that metal-phenolic network-based nano-coating made from iron (III) and tannic acid can help protect probiotic microbes from the detrimental effect of antibiotics (Ashraf et al., 2023; Guo and Wu, 2023). Due to their physicochemical parameters, smaller structures, and thermodynamic properties, nanoliposomes enjoy vast applications for a wide range of products. The stability of *L. rhamnosus* was analyzed by loading them into chitosan-gelatin coated nanoliposomes. The characterization study suggested the successful coating of bifidobacteria with coated nanoliposomes. Further supported by the results of simulated GI fluids with a significant amount of viable cells present in the fluid guiding toward the suitability of nanoliposomes as a potential carrier of probiotics in developing nutraceutical foods (Hosseini et al., 2022).

## 6. Conclusion

Probiotics have well-documented physiological effects with a definitive mechanism. However, the exact mechanism of how they work to enhance health and prevent different diseases must be explored. Evidence from well-documented clinical trials has revealed that probiotics can potentially alleviate different GI and other disorders. Despite our understanding of some molecular mechanisms underlying beneficial aspects of probiotics, we are still far from clinically proven efficacy in many autoimmune and inflammatory diseases. Moreover, many studies have been done on the animal model, so there is an emergent need to translate these results into humans. Currently, genetically modified commensal lactic acid bacteria are being used to deliver special health-interest compounds. But most of the work regarding recombinant bacteria is related to vaccines. However, genetically modified bacteria can be used for exploring innovative strategies to deliver bioactive molecules to mucosal tissues. More consistent and reproducible clinical trials are required to reveal probiotics efficacy, limitations, and safety, determining their effects on the immune system. Considering all the methodologies discussed in this review, probiotics can be applied easily by food producers to make novel functional foods to promote human health.

## Author contributions

All authors wrote the manuscript, read and agreed to the published version of the manuscript.

## Funding

This work was based upon the work from COST Action 18101 SOURDOMICS—Sourdough biotechnology network toward novel, healthier, and sustainable food and bioprocesses (https://sourdomics.com/; https://www.cost.eu/actions/CA18101/, accessed on 12 April 2023), where TE was member of the working groups 4, 6, 7, and 8, FÖ was the leader of the working group 8, “Food safety, health-promoting, sensorial perception and consumers’ behavior” and JR was the Chair and Grant Holder Scientific Representative and is supported by COST (European Cooperation in Science and Technology) (https://www.cost.eu/, accessed on 12 April 2023). COST was a funding agency for research and innovation networks. JR also acknowledged the Universidade Católica Portuguesa, CBQF—Centro de Biotecnologia e Química Fina—Laboratório Associado, Escola Superior de Biotecnologia, Porto, Portugal, as well as the support made by LA/P/0045/2020 (Alice) and UIDB/00511/2020-UIDP/00511/2020 (LEPABE) funded by national funds through FCT/MCTES (PIDDAC).

## Conflict of interest

The authors declare that the research was conducted in the absence of any commercial or financial relationships that could be construed as a potential conflict of interest.

## Publisher’s note

All claims expressed in this article are solely those of the authors and do not necessarily represent those of their affiliated organizations, or those of the publisher, the editors and the reviewers. Any product that may be evaluated in this article, or claim that may be made by its manufacturer, is not guaranteed or endorsed by the publisher.
