# Corrigendum: Probiotics: mechanism of action, health benefits and their application in food industries

**DOI:** 10.3389/fmicb.2024.1378225

**Published:** 2024-02-14

**Authors:** Anam Latif, Aamir Shehzad, Sobia Niazi, Asna Zahid, Waqas Ashraf, Muhammad Waheed Iqbal, Abdur Rehman, Tahreem Riaz, Rana Muhammad Aadil, Imran Mahmood Khan, Fatih Özogul, João Miguel Rocha, Tuba Esatbeyoglu, Sameh A. Korma

**Affiliations:** ^1^Department of Human Nutrition and Dietetics, School of Food and Agricultural Sciences, University of Management and Technology, Lahore, Pakistan; ^2^UniLaSalle, Univ. Artois, ULR7519 - Transformations & Agro-resources, Normandie Université, Mont-Saint-Aignan, France; ^3^State Key Laboratory of Food Science and Technology, Jiangnan University, Wuxi, China; ^4^National Institute of Food Science and Technology, University of Agriculture, Faisalabad, Pakistan; ^5^School of Food Science and Technology, Jiangnan University, Wuxi, Jiangsu, China; ^6^School of Food and Biological Engineering, Jiangsu University, Zhenjiang, Jiangsu, China; ^7^College of Food and Biological Engineering, Jimei University, Xiamen, China; ^8^Department of Seafood Processing Technology, Faculty of Fisheries, Cukurova University, Adana, Türkiye; ^9^Biotechnology Research and Application Center, Cukurova University, Adana, Türkiye; ^10^CBQF - Centro de Biotecnologia e Química Fina – Laboratório Associado, Escola Superior de Biotecnologia, Universidade Católica Portuguesa, Porto, Portugal; ^11^LEPABE—Laboratory for Process Engineering, Environment, Biotechnology and Energy, Faculty of Engineering, University of Porto, Porto, Portugal; ^12^ALiCE—Associate Laboratory in Chemical Engineering, Faculty of Engineering, University of Porto, Porto, Portugal; ^13^Department of Food Development and Food Quality, Institute of Food Science and Human Nutrition, Gottfried Wilhelm Leibniz University Hannover, Hannover, Germany; ^14^Department of Food Science, Faculty of Agriculture, Zagazig University, Zagazig, Egypt; ^15^School of Food Science and Engineering, South China University of Technology, Guangzhou, China

**Keywords:** probiotics, lactic acid bacteria, immunomodulation, anti-allergic and gastrointestinal diseases, functional foods

In the published article, there was an error in affiliation 2. Instead of “UniLaSalle, Transformations and Agroressources Research Unit, Mont-Saint-Aignan, France”, it should be “UniLaSalle, Univ. Artois, ULR7519 - Transformations & Agro-resources, Normandie Université, Mont-Saint-Aignan, France.”

The authors apologize for this error and state that this does not change the scientific conclusions of the article in any way. The original article has been updated.

